# Tying the knot: The cystine signature and molecular-recognition processes of the vascular endothelial growth factor family of angiogenic cytokines

**DOI:** 10.1111/j.1742-4658.2011.08350.x

**Published:** 2011-11

**Authors:** Shalini Iyer, K Ravi Acharya

**Affiliations:** Department of Biology and Biochemistry, University of BathClaverton Down, Bath, UK

**Keywords:** angiogenesis, cystine-knot growth factors, molecular recognition, vascular endothelial growth factor (VEGF) family, VEGF receptors

## Abstract

The cystine-knot motif, made up of three intertwined disulfide bridges, is a unique feature of several toxins, cyclotides and growth factors, and occurs in a variety of species, including fungi, insects, molluscs and mammals. Growth factor molecules containing the cystine-knot motif serve as ligands for a diverse range of receptors and play an important role in extracellular signalling. This superfamily of polypeptides comprises several homodimeric and heterodimeric molecules that are central characters in both health and disease. Amongst these molecules are a group of proteins that belong to the vascular endothelial growth factor (VEGF) subfamily. The members of this family are known angiogenic factors that regulate processes leading to blood vessel formation in physiological and pathological conditions. The focus of the present review is on the structural characteristics of proteins that belong to the VEGF family and on signal-transduction pathways that become initiated via the VEGF receptors.

## Introduction

**Fig. 1:**
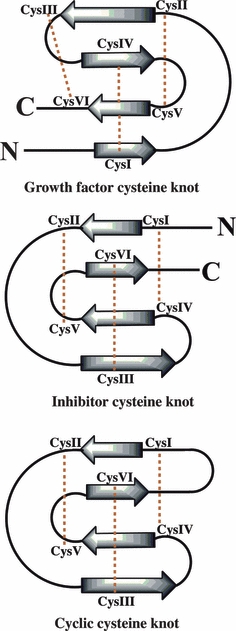
Schematic representation of the three groups of cystine-knot proteins [107]: GFCKs, ICKs and CCKs. The cysteine residues are labelled I to VI and the disulfide bonds are shown as dotted lines.

Disulfide bonds between pairs of cysteine residues in some proteins form a unique functional signature and are considered to be major determinants of protein stability and folding. The cystine-knot motif, formally identified as a structural motif about 20 years ago [1], is one such arrangement of disulfide bonds that is present in peptides and proteins from a wide variety of species. This knotted arrangement of disulfide bridges is usually associated with β-sheet structures in proteins in which they occur. Although the motif was initially thought to be characteristic of some growth factors, it soon became apparent that the cystine-knot is also quite common in a variety of smaller peptides, especially the small cysteine-rich toxins. However, when the spatial properties of the knot in these smaller peptides were taken into account, the motif could not be superimposed directly with those of the growth factors. This led to the classification of the cystine-knot-containing proteins into three groups ([2]; Fig. 1): growth factor cystine-knots (GFCKs), inhibitor cystine-knots (ICKs) and cyclic cystine-knots (CCKs). The disulfide connectivity in all these cystine-knot molecules is identical. Usually six cysteine residues, labelled in order from the N-terminus to the C-terminus, are involved in forming the knot. The knot is an embedded ring formed by two disulfide bridges and their connecting backbone segments, which is penetrated by a third disulfide bridge. The three disulfide bridges are formed between Cys I and Cys IV, Cys II and Cys V and Cys III and Cys VI (any intervening cysteine not involved in the formation of a disulfide bond is conventionally ignored from being labelled). The main distinguishing feature between these three families is that the penetrating disulfide bridge for the GFCKs is Cys (I–IV) while that for the other two families is Cys (III–VI). The unique feature that distinguishes the CCKs from the ICKs is the cyclic nature of their protein backbone. One of the interesting features of cyclic peptides is that knowledge of the peptide sequence does not reveal the ancestral head and tail; knowledge of the gene sequence is required for this. Post-translational linkage of the N-terminal and C-terminal residues via a peptide bond results in cyclisation of this molecule.

## Growth factor cystine-knots

**Fig. 2:**
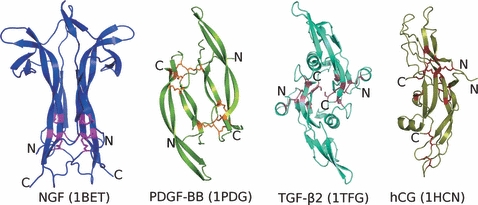
Ribbon representation of the four GFCK prototypes [108]. The cystine-knot motif is shown in all four structures as a ball-and-stick model. The PDB codes for coordinates used are indicated in parentheses.

The cystine-knot superfamily of growth factors is a diverse group of proteins that are involved in a range of biological functions such as cell growth, organogenesis, embryonic development, cell-to-cell communication and differentiation, as well as tissue repair and remodelling. In addition to being involved in many of the normal physiological functions of the cell, these growth factors are also known to influence the pathophysiological outcome of several malignant disorders. GFCKs can be further divided into four groups; each subfamily is exemplified by prototypes such as transforming growth factor-β (TGF-β), nerve growth factor (NGF), platelet-derived growth factor (PDGF) and glycoprotein hormones (GPHs) such as gonadotropins (Fig. 2). In addition to these well-known members, novel proteins are being continuously added to the growing repertoire that is the cystine-knot superfamily [3]. These new members are collectively called the C-terminal cystine-knot (CTCK) proteins. The proteins of this subfamily are functionally diverse modular proteins that share a conserved domain of about 90 amino-acid residues in their C-terminal cysteine-rich region. Members of the CTCK family include von Willebrand Factor, mucins, Cyr61 [cysteine-rich protein 61 (CCN)] proteins [4], Slit protein and the Norrie Disease Protein. Phylogenetic analysis [3] of the cystine-knot-containing proteins identified two main branches: the TGF-β (1TFG) family forms one definite group and the PDGF (1PDG), NGF (1BET) and GPH (1HCN) families form the other main branch of the phylogenetic tree.

Included in the PDGF family of cytokines is a subgroup of growth factors that are encoded by several genes which regulate the processes of angiogenesis and lymphangiogenesis. This family of polypeptides is known as the vascular endothelial growth factor (VEGF) family of cystine-knots. This review focuses on the structural homology shared by these growth factors by virtue of the cystine-knot motif and how the knot-like topology has played an important role in receptor recognition and signalling transduction events that lead to blood and lymphatic vessel development.

## VEGF family: ligands

**Fig. 3:**
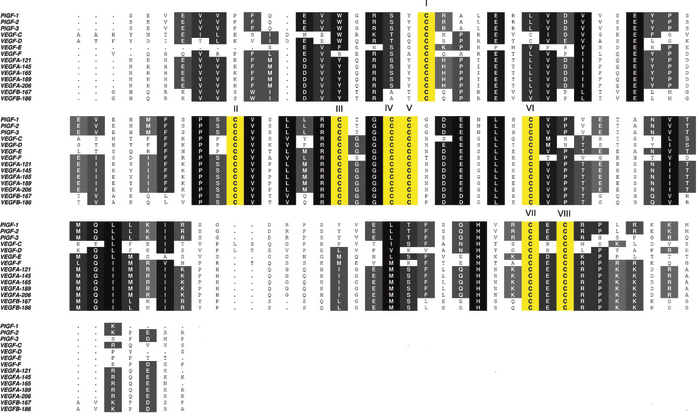
Sequence alignment of all human VEGF family proteins. The alignment was created using the program ALINE [109]. The amino acid residues have been coloured based on similarity. Identical residues are shaded black and residues of similar character are coloured in shades of grey. The cysteine residues that are involved in the formation of the knot are shaded yellow. They are numbered in order from N-terminus to the C-terminus. The three disulfide bridges are formed between Cys I and Cys VI, Cys III and Cys VII, and Cys V and Cys VIII.

**Fig. 4:**
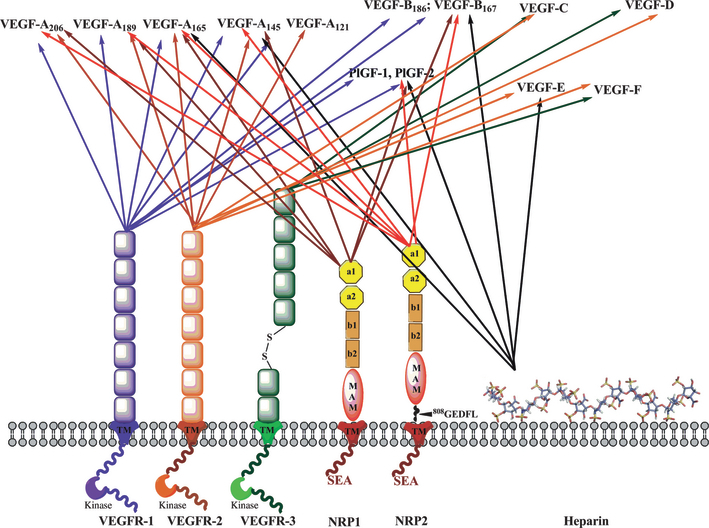
A schematic representation of the various receptors characterized for the members of the VEGF family of cystine-knot proteins [107]. VEGFR-1 (fetal murine sarcoma-like tyrosine kinase (Flt)-1), VEGFR-2 [kinase-insert domain receptor (KDR)] and VEGFR-3 (Flt-4) are tyrosine kinase receptors. NRP-1 and NRP-2 (neuropilins) belong to the family of semaphorin receptors. HSPGs act as binding partners for some of the isoforms of the VEGF proteins. The receptors have been coloured individually. The arrows point to the ligands that bind each of these receptors and these have been coloured according to the receptor they represent.

The VEGF family comprises six subgroups of proteins: VEGF-A, -B, -C, -D and -E and placenta growth factor (PlGF). These bioactive proteins exemplify the development of distinct biological functionalities during the process of blood vessel formation. These polypeptides display a common structural architecture despite little sequence homology (Fig. 3). The crystal structures of these growth factors demonstrate that all have a similar topology based on the cyclic-knot of cysteines involved in both intrachain and interchain disulfide bonds. This cysteine connectivity stabilizes the Cα framework of these growth factors for elaboration of the solvent-exposed loop regions that form the receptor-binding surface on these polypeptides. The members of this family are biologically active as dimers: mainly as homodimers and sometimes as heterodimers. These growth factors mediate their different biological roles by binding to three high-affinity tyrosine kinase receptors (Fig. 4): vascular endothelial growth factor receptor (VEGFR)-1, VEGFR-2 and VEGFR-3 [5–7]. VEGFR-1 and VEGFR-2 have differential kinase activation potentials, although both are important for normal development. VEGFR-1 and VEGFR-2 are primarily involved in blood vessel growth, whereas VEGFR-3 is mainly involved in haematopoiesis and lymphangiogenesis. Apart from these three tyrosine kinases, different splice forms of VEGF-A, VEGF-B and PlGF bind the semaphorin receptors neuropilins (NRPs) 1 and 2 [8]. VEGF-E (the viral VEGF) has also been shown to bind NRP-1, even though it lacks a heparin-binding domain. Some of these isoforms also bind heparan sulfate proteoglycans (HSPGs). VEGFs and VEGFRs are also known to form complexes with integrins and extracellular matrix components.

### Vascular endothelial growth factor-A

**Fig. 5:**
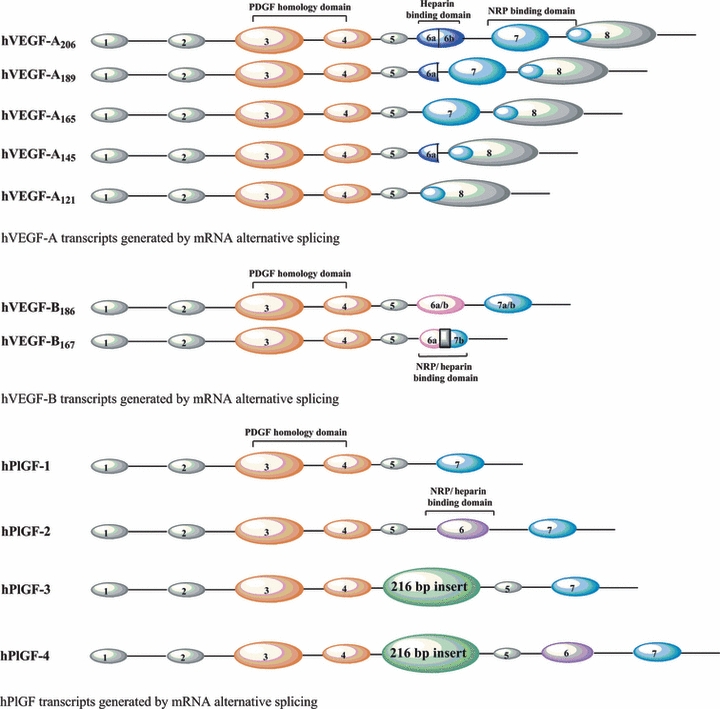
Genomic (human, denoted with the prefix ‘h’) organization and alternative splice forms of VEGF-A, VEGF-B and PlGF [107]. The exons coding for the PDGF homology domain (orange), heparin-binding domain (dark blue) and NRP-binding domain (light blue) are indicated in the figure. The exons are shown as oval, shaded structures, whereas the introns are represented by lines. The sizes and lengths of the exons and introns have not been drawn to scale.

VEGF-A is the most potent and pivotal regulator of angiogenesis and vasculogenesis. A highly specific mitogen for vascular endothelial cells (ECs), VEGF-A promotes extravasation of proteins from tumour-associated blood vessels. Disruption of genes encoding VEGF-A results in severe defects and abnormalities in the development of the cardiovascular system. Hypoxia is one of the major up-regulators of VEGF-A expression and is thought to drive angiogenesis during organogenesis [9]. On the other hand, limited/reduced VEGF-A supply to the tissues leads to inhibition of organ development [10,11]. Alternative splicing of *VEGF-A* mRNA produces five isoforms: VEGF-A_121_, VEGF-A_145_, VEGF-A_165_, VEGF-A_189_ and VEGF-A_206_ (Fig. 5). These isoforms differ in their heparin and heparan sulfate-binding abilities.

VEGF is expressed in spatial and temporal association with physiological as well as tumour angiogenesis *in vivo*. Expression of VEGF-A induces the formation of vesiculo-vacuolar organelles that channel the blood-borne proteins into the tumours. This forms an extravascular fibrin gel that stimulates ECs and tumour cells to proliferate and migrate and also supports the invasion of stromal cells into the growing tumours [12]. The similar mechanism of VEGF-A induction during physiological as well as tumour angiogenesis explains why VEGF-A plays a central role in so many types of diverse tumours.

VEGF-A expression can be stimulated by several other factors, even in the presence of oxygen. Activated oncogenes that are part of the ras/mitogen-activated protein kinase (MAPK) signal transduction pathway potentiate expression of *VEGF-A* mRNA [13]. Hypoxia-independent production of VEGF-A can also be brought about by hypoglycaemia [14] and by inactivation of tumour-suppressor genes such as von Hippel Landau and p53 genes at both transcriptional and post-transcriptional levels [15,16].

Inhibition of VEGF-A signalling inhibits the development of many tumours. The production of antagonistic VEGF-A mutants, VEGF receptor inhibitors, antisense mRNA-expressing constructs, inhibitory soluble receptors and humanized monoclonal antibodies against human VEGF-A are some of the strategies that are being undertaken to treat VEGF-induced tumour angiogenesis. VEGF-A is also being used to develop therapeutics for the treatment of diseases related to dysfunctional angiogenesis [17,18].

Apart from its role as an endothelial-specific factor, VEGF-A has also been classified as an angioneurin because it is known to affect both vascular cell and neural cell functions. Recent studies have shown that VEGF-A has an important role to play in vessel and neuronal wiring in the central nervous system (CNS). VEGF-A, through its interaction with VEGFR-2, induces vascularization of the neural tube via the formation of a perineural vascular plexus [19]. It has been shown that a spatial gradient formed by the different isoforms of VEGF-A is essential for proper vessel patterning in the brain [20]. VEGF-A also regulates neuronal cell migration by interacting with NRP-1 [21]. Besides its effects on synaptic plasticity in the CNS and neuroprotective effects on different neuronal cell types in both the CNS and the peripheral nervous system, it has also been shown that there is strong evidence for an unsuspected link between VEGF-A and motor neurons from the studies of amyotrophic lateral sclerosis (ALS) [22,23]. Apart from ALS, VEGF-A has been linked to several other neurodegenerative diseases such as Alzheimer's disease [24] and Parkinson's disease [25], and to neuropathies such as those associated with diabetes, ischemia and nerve injury.

### Vascular endothelial growth factor-B

VEGF-B, like VEGF-A, occurs in alternately spliced forms (Fig. 5): VEGF-B_167_ and VEGF-B_186_ [26,27]. VEGF-B is both structurally and functionally related to VEGF-A and PlGF. The smaller isoform is highly basic and is associated with the cell surface via HSPGs. VEGF-B controls the bioavailability of VEGF-A by forming heterodimers with it. Both isoforms of VEGF-B are devoid of N-glycosylation sites, although VEGF-B_186_ has an O-glycosylation site instead [26].

VEGF-B has wide tissue distribution, albeit overlapping with VEGF-A, and experiments reveal that VEGF-B can act as an EC growth factor [26]. VEGF-B displays quite prominent expression in the developing heart and in several muscle types during embryonic development [28]. Gene-knockout studies performed in mice by ablating VEGF-B expression revealed that response to myocardial recovery from ischemia and vascular occlusion is jeopardized [29]. Studies show that VEGF-B_167_ (along with VEGF-A_165_ and PlGF-1) can induce mast cell chemotaxis and has a role to play in inflammatory and neoplastic angiogenesis [30]. VEGF-B has also been implicated in several pathological conditions, such as metastases of cancer cells, through activation of plasminogen activator, pulmonary hypertension and growth of tumours [31]. Interestingly, some recent studies implicate a role for VEGF-B in lipid metabolism, a function not yet assigned for an angiogenic growth factor [32].

### Vascular endothelial growth factor-C

**Fig. 6:**
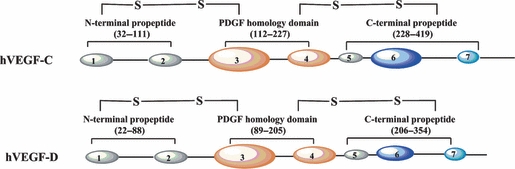
Genomic (human) organization of VEGF-C and VEGF-D precursor proteins [107]. The PDGF homology domain (shown in orange) in the precursor proteins is covalently linked to the N-terminal and the C-terminal pro-peptides via disulfide bonds (S-S). The amino acid positions for each of the three proteolytically processed domains are shown in parentheses.

The VEGF homology domain in VEGF-C is flanked by unique N- and C-terminal extensions (Fig. 6). The carboxy-terminal domain contains a repetitive pattern of cysteine residues, Cys–X_10_–Cys–X–Cys–X–Cys. The pro-peptide VEGF-C undergoes stepwise proteolytic cleavage to form a 21-kDa protein [27,33]. Interestingly, VEGF-C has been described to occur as a mixture of covalently and noncovalently bound dimers [34,35]. VEGF-C is expressed during embryonic development in regions where lymph vessels sprout from blood vessels. In adults, however, VEGF-C is mainly restricted to the lymphatic endothelium and has been implicated in the development of lymphatic vessels [36,37]. C-terminally cleaved VEGF-C is a high-affinity ligand for VEGFR-3 and upon removal of both pro-peptides it acquires binding affinity for VEGFR-2. It has a higher binding affinity for VEGFR-3 than for VEGFR-2. Unlike some of the other VEGF family members, VEGF-C does not bind heparin. It selectively stimulates lymphangiogenesis in the chorioallantoic membrane, whereas VEGF-A promotes haematic proliferation [33].

### Vascular endothelial growth factor-D

VEGF-D is yet another member of the VEGF family of proteins. It shares around 48% amino-acid sequence identity with VEGF-C [38]. Like VEGF-C, the N-terminal and C-terminal pro-peptides (Fig. 6) of VEGF-D also undergo stepwise proteolytic cleavage to acquire binding affinity for VEGFR-2 and VEGFR-3 [39]. VEGF-D is a tumour angiogenesis factor and promotes EC proliferation. Experiments with a mouse tumour model reveal that VEGF-D stimulates lymphangiogenesis within tumours. It promotes tumour metastasis via development of the lymph vessels by activating VEGFR-3 and not VEGFR-2 [40]. VEGF-D is also known to play a modifier role by modulating the abundance of lymphatic vessels in some tissues during embryonic development [41,42]. In adults, VEGF-D is localized in smooth muscle cells in a variety of tissues, suggesting that it might play a role in facilitating rapid repair of vessels in the event of tissue damage [43]. VEGF-D has also been implicated in mechanisms of resistance to clinical anti-angiogenic agents. It has been suggested that because VEGF-D is an alternative ligand to VEGF-A for VEGFR-2, VEGF-D could be potentially responsible for patients not responding, or developing resistance, to Avastin [44].

### Vascular endothelial growth factor-E

Pox viruses of the Orf family encode reading frames for proteins called VEGF-E that show only 25-35% amino-acid identity with VEGF-A (Fig. 3) but bind with comparable affinity to VEGFR-2 [45]. VEGF-E isoforms display a considerable degree of sequence variation [46]. They all lack a heparin-binding domain, but some variants retain binding to NRP-1 [47]. The viral VEGFs are potent mitogens that stimulate proliferation of human ECs *in vitro* and vascularization of sheep skin *in vivo* with potencies equivalent to that of VEGF-A [48]. It was also shown that transgenic mice over-expressing the NZ7 variant of VEGF-E showed increased vascularization in subcutaneous tissue without producing the oedematous lesions typically present on the skin of VEGF-A transgenic mice. Studies by Kiba *et al.* [49] showed that exchanging the region encompassing loops L1 and L3 of the VEGF-E variant NZ7 with the corresponding loops from VEGFR-1-binding ligands, such as PlGF or VEGF-A, strongly reduced the activity of this viral VEGF, implying specific interactions between L1 and L3, and VEGFR-2.

### Vascular endothelial growth factor-F

VEGF-Fs are the snake-venom-derived VEGFs, vammin and VR-1. These VEGF homologues possess < 50% amino-acid sequence identity (Fig. 3) with VEGF-A_165_ [50]. The two VEGF-F proteins display potent biological activities both *in vitro* and *in vivo* when compared with VEGF-A_165_. VEGF-Fs are 25-kDa homodimeric heparin-binding proteins with a markedly short C-terminal region (of 16–17 residues). This region does not bear any significant homology to the C-terminal region of other VEGF homologues. It was shown that VEGF-F binds heparin-like molecules via its C-terminal region and inhibits the biological activity of VEGF-A_165_ [51].

### Placenta growth factor

The human term placenta codes for a VEGF homologue known as the PlGF [52]. An alternative splicing mechanism produces four isoforms of PlGF, numbered 1–4 (Fig. 5). *PlGF* mRNA is abundantly expressed in placental tissue and is also present in very small amounts in heart, lung, thyroid, goitre and skeletal muscle. No expression of PlGF has been detected in kidney and pancreas [53].

Hybridization studies have revealed that PlGF is well conserved in the mammalian, bovine, chicken, amphibian and insect genomes. This suggests that the *PlGF* gene has specific and indispensable functions. The importance of this protein is emphasized by the fact that a dysfunctional/absent gene in a mouse embryo leads to an impaired blood vessel network and subsequently to the death of the embryo. The development and cell-specific regulation of the process of alternative mRNA splicing may have important consequences for physiological and pathological processes. Studies reveal a preferential expression of PlGF-2 in the placental tissue and cell lines, suggesting that this long form of PlGF may be involved in the growth and maintenance of pregnancy [54].

**Fig. 7:**
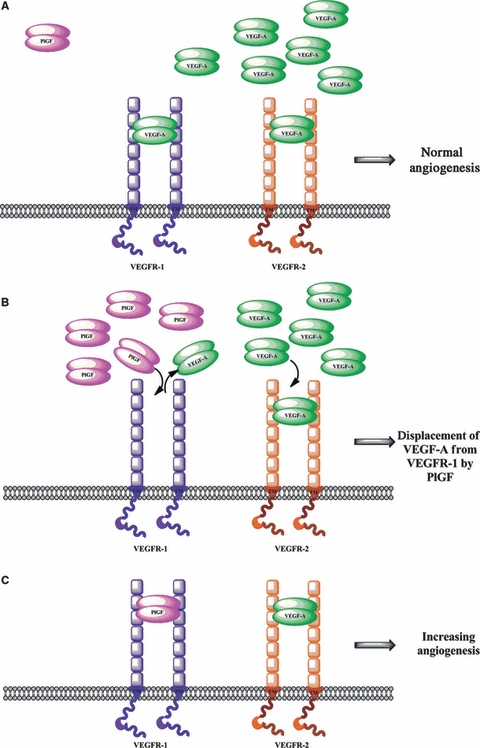
Modification of the EC response to VEGF-A (green) by PlGF (magenta) during pathological conditions [107]. When the concentration of PlGF is low, both VEGFR-1 (purple) and VEGFR-2 (orange) bind VEGF-A and normal angiogenesis occurs (A). However, during pathological conditions (B and C), there is an increase in the concentration of PlGF, which displaces VEGF-A from VEGFR-1 and thereby increases the bioavailability of VEGF-A for VEGFR-2 [59].

PlGF is also induced in human keratinocytes during wound healing [55], its expression regulated by key cytokines released during an injury or a wound. It has also been shown that melanoma progression in humans is accompanied by deregulated, constitutive PlGF expression. PlGF, however, serves no apparent autocrine role in melanoma proliferation. It has been established that recombinant, purified PlGF-2 is able to stimulate bovine aortic ECs and HUVECs [56], but not the ECs from hepatic sinusoids [57]. PlGF isoforms have very little mitogenic or permeability-enhancing activity. However, they significantly potentiate the action of low concentrations of VEGF-A *in vitro* and more strikingly *in vivo* [58]. Gene knockout studies by Carmeliet *et al.* [59] revealed that synergistic cooperation between PlGF and VEGF-A in pathological conditions is specific (Fig. 7). Upregulation of PlGF by ECs leads to displacement of VEGF-A from its receptor, thus increasing the bioavailability of VEGF-A [60].

## VEGF family: three-dimensional structures and functional implications

The aim of angiogenesis research is to characterize the ECs that originate from tumour tissues, to continue to identify biochemical targets and elucidate the three-dimensional (3D) structures of all the macromolecules involved in the process of angiogenesis. It is important to elucidate and quantify the structure–function relationship of these macromolecules in parametric terms in order to correlate the dynamics of all biological processes and unravel the complex pathways that are integrated into the process of blood vessel formation. The 3D structures define the interface of ligands and their receptors or other macromolecular targets. Structural studies on the VEGF family of proteins and their receptors have been the focus of much research. The following section discusses the insights gained into the function of these angiogenic factors by the study of their structures: native as well as in complex with their cognate receptors or antibodies generated specifically to abolish ligand–receptor interactions.

### Native structures: unbound forms

**Fig. 8:**
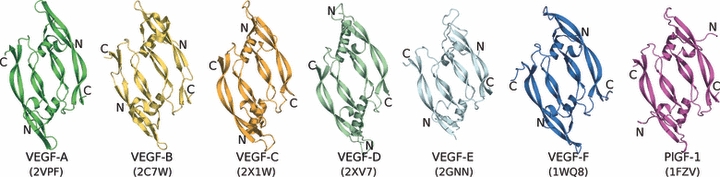
Ribbon representation of the members belonging to the VEGF family of growth factors [108]. VEGF-A is coloured green (2VPF; [61]), VEGF-B is coloured orange-yellow (2C7W; [63]), VEGF-C is coloured bright orange (2X1W; [74]), VEGF-D is coloured pale green (2XV7; [64]), VEGF-E is coloured pale cyan (2GNN; [65]), VEGF-F is coloured dark blue (2WQ8; [50]) and PlGF-1 is coloured magenta (1FZV; [62]).

To date, crystal structures of the unbound forms of the receptor-binding domains of VEGF-A [61], PlGF-1 [62] and VEGF-B [63] and VEGF-D [64], VEGF-E [65] and VEGF-F [50] have been reported. All six structures are remarkably similar to each other in their topology, with distinct structural differences in the N-terminal region (Fig. 8). A high degree of sequence conservation of the structurally important residues between these growth factors results in the same overall 3D structure being adopted. VEGF-D has an extended N-terminal α-helix, which in the other three structures is short with the preceding residues folding away from the receptor-binding interface. The 3D structures of these proteins is fashioned by a nonglobular sheet-like structure made up of highly twisted antiparallel β-strands with two distorted β-hairpin loops on one side of the cystine-knot and a single loop on the other. VEGF proteins occur as biological homodimers in which the monomers associate via hydrophobic interactions with the dimer axis perpendicular to the plane of the β-sheet. Each monomer consists of two α-helices and seven β-strands. There are very few contacts between the two monomers at the central, highly irregular and solvent-accessible β-sheet region. This highly twisted (the largest twist is observed in strand β4) and antiparallel β-sheet region of the growth factors superposes well, even to the extent of maintaining some of the important hydrogen-bonding donor and acceptor residues.

The dimeric structure is stabilized by the cystine-knot motif and a hydrophobic core region (one per monomer). The cystine-knot is formed by the first two bonds and the backbone of the intervening polypeptide through which the third disulfide bridge passes. The cystine-knot motif in the VEGF family of growth factors also consists of two interchain disulfide bonds that hold the two monomers together. A conserved gap size between the cysteines in the knot ensures that their positions in the β-strands are maintained amongst all VEGF proteins. All the cysteines involved in forming the knot (including those involved in forming the interchain disulfide bond) have their main-chain torsion angles (ϕ, ψ) within the range specified for a typical β-strand. Conservation of a glycine residue at the position that precedes the cysteine residue involved in the formation of an interchain disulfide bond emphasizes the importance of the motif in maintaining structural integrity of the proteins belonging to this superfamily. This glycine has been seen to maintain positive dihedral main-chain conformation in all the structures solved for the different VEGF proteins.

The cystine core provides excellent proteolytic resistance and thermal stability to these growth factors. Thermodynamic stability and thermal-stability analyses of different disulfide mutants of VEGF-A, performed by Muller *et al.* [66], showed that the cystine-knot is responsible for entropic stabilization of the molecule and that none of the disulfide bridges increases the thermodynamic stability of VEGF-A. Crystal structures of the disulfide mutants revealed that the structural differences in these mutants were restricted to differences in loop structures or differences in the coplanar arrangement of the monomers in the dimer. Although a similar thermodynamic characterization has not been carried out for any of the other members of the VEGF family, it is very likely that the other members emulate similar thermodynamic properties.

### Receptor complexes: bound forms

**Fig. 9:**
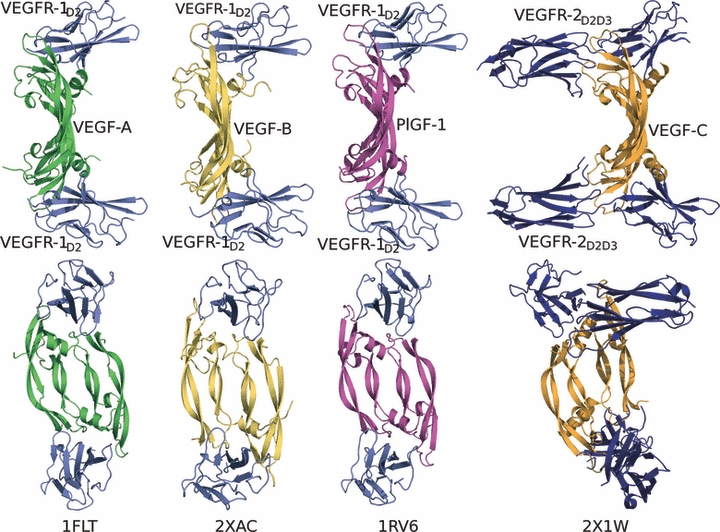
Ribbon representation of the different receptor–growth factor complexes [108]. The top panel shows the side view of the complex while the bottom panel shows the front view. The receptor and the growth factor molecules have been labelled. VEGFR-1 is shown in light blue, whereas VEGFR-2 is coloured dark blue. VEGF-A is coloured green, VEGF-B is coloured orange-yellow, VEGF-C is coloured bright orange and PlGF-1 is coloured in magenta. The structures shown are VEGF-A_8–109_•VEGFR-1_D2_ complex (1FLT; [67]), VEGF-B_10–108_•VEGFR-1_D2_ complex (2XAC; [72]), PlGF-1•VEGFR-1_D2_ complex (1RV6; [73]), and VEGF-C•VEGFR-2_D2D3_ complex (2X1W; [74]).

A considerable amount of structural information is available for VEGF-A complexes. Three-dimensional structures are available for VEGF-A in complex with VEGFR-1 [67], Fab-12 [68], Fab-Y0317 [69], Fab-G6 and Fab-B20-4 [70]. The same is not true for the VEGFR-1-specific ligands VEGF-B and PlGF. Structural data on VEGF-B (apart from the native form) include the structure of this ligand in complex with a neutralizing antibody fragment, Fab2H10 [71] and in complex with VEGFR-1 [72]. Only the complex of PlGF with VEGFR-1 [73] has been reported to date. Recently, the structure of VEGF-C was elucidated in complex with VEGFR-2 [74] (Fig. 9).

The topology of the receptor–ligand complex is essentially the same amongst the VEGF family of ligands. Two receptor molecules ligate with the growth factor, one at each of the two symmetrical ends of the dimer formed by virtue of two intersubunit disulfide bridges. This mode of antiparallel dimerization brings into spatial proximity residues (from both monomers) that are involved in binding receptors. Another striking feature of dimerization is the clustering of positive amino acids (for some of the isoforms of the different VEGF proteins). This region is believed to play an important role in binding HSPGs and therefore enhances their receptor-binding affinity. Pairwise superposition of these dimeric growth factors in their receptor-bound state versus their native form results in very low C^α^ displacement values, indicating that the growth factors do not undergo any major conformational change and require no induced-fit mechanism to enable binding to their respective partners. However, superposition of the C^α^ traces of these growth factors reveals conformational differences at the N-terminal region, some loop regions and the C-terminal region. Interestingly, these loop regions form part of the receptor-binding interface. These variable regions are responsible for the functional differences observed between the VEGF proteins. The compact and stable framework provided by the cystine-knot confers upon these growth factors the ability to recognize common binding partners, whereas the variability of the loop regions allows for the presentation of active residues for specific binding interactions, which lead to a diverse range of biological functions mediated by the VEGF proteins. The description that follows focuses mainly on the different VEGFR-1 complexes elucidated so far, with some discussion on the similarities and differences with the VEGFR-2 complex [74].

The interface between VEGFR-1 and its ligands is flat, largely hydrophobic and hence energetically favoured by shape complementarity between the two interacting surfaces. The two interfaces at each end of the growth factor dimer are usually identical, with the exception of loss/gain of a couple of residues from both the receptor and the ligand. The structures of the three VEGFR-1 complexes reveal that several negatively charged residues seem to mediate receptor binding to some degree, although the contributions to binding by these acidic residues (Asp63, Glu64 and Glu67 from VEGF-A) seem to be essential for the interaction of VEGF-A and PlGF with VEGFR-1 [75,76] but not so much for VEGF-B [77]. The major VEGFR-1-binding determinants on VEGF-B are yet to be established. Analysis of the amino-acid sequence of VEGF-A, VEGF-B and PlGF shows that despite low sequence homology within the residues interacting with the receptor, the binding stretch is almost identical in all three complexes. Although only 33% of the total residues contributed by the ligands to the binding interface are conserved, the amino acids compensate for the differences in the sequence between these three members of the VEGF family by occupying structurally equivalent positions and mediating very similar interactions with the receptor. On the other hand, the contribution of VEGFR-1 to the binding interface remains unaltered because the residues presented to the interface by the receptor are identical in all three complexes. Most of these residues are involved in interacting with their respective ligands via hydrophobic or hydrogen-bonding interactions. However, important differences were observed when the surface areas of these residues, accessible after ligand binding, were analysed [72]. We hypothesize that these differences in surface accessibility might play a seminal role in deciding the importance of these residues in the affinity of these ligands towards VEGFR-1.

The structure of VEGF-C in complex with VEGFR-2 [74] revealed that the overall topology of the VEGFR-2 complex was essentially the same as the previously elucidated VEGFR-1 complexes. VEGF-C has the same topological fold and binds its receptor in a manner similar to the other members of the VEGF family, and the domain 2 of VEGFR-2 is structurally similar to the domain 2 of VEGFR-1 with which it shares about 32% amino acid sequence identity. This architectural similarity is, however, not carried through to the residues that interact with the growth factors in their respective receptor complexes. The degree of dissimilarity was shown to increase when the electrostatic potential of the binding interface in all the complexes was taken into consideration [72]. The VEGFR-1 interface, which is mainly basic, and the VEGFR-2 interface, which is mainly negatively charged, supports the specificity and receptor recognition profile of the different members of the VEGF family.

**Fig. 10:**
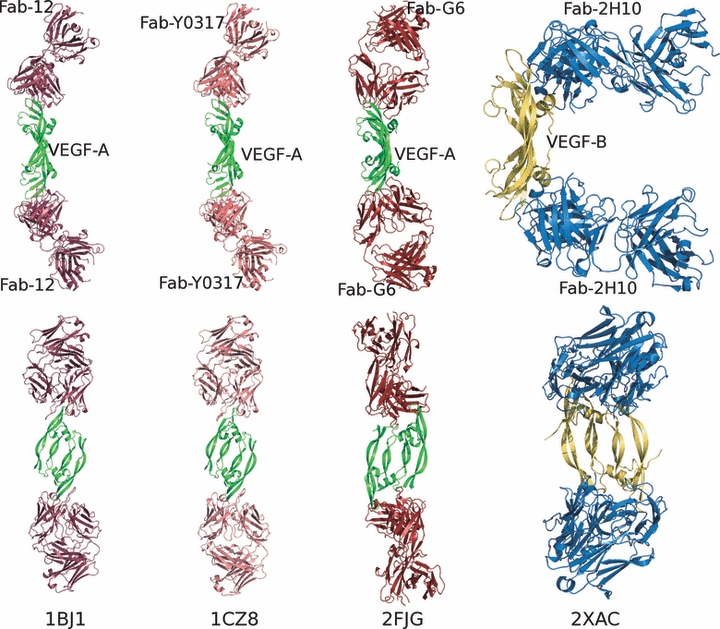
Ribbon representation of the different antibody–growth factor complexes [108]. The top panel shows the side view of the complex while the bottom panel shows the front view. The antibody and the growth factor molecules have been labelled. The different generations of the VEGF-A-binding Fab molecules are shown in different shades of red, whereas Fab-binding VEGF-B is coloured marine blue. VEGF-A is coloured green and VEGF-B is coloured orange-yellow. The structures shown are of the complexes of VEGF-A_8–109_ with Fab-12 (1BJ1; [68]), Fab-Y0317 (1CZ8; [69]) and Fab-G6 (2FJG; [70]), and of VEGF-B with Fab-2H10 (2VWE; [71]).

Apart from these receptor complexes, several structures of antibody complexes have also been elucidated. Most of these are structures of VEGF-A in complex with different antibodies (Fig. 10). The structure of VEGF-B in complex with a humanized antigen-binding fragment prepared from a murine monoclonal antibody has also been elucidated [71]. The most notable difference between the two types of complexes lies in the character of residues that mediate the interactions. The structures show that receptor-bound complexes favour nonpolar interactions, whereas uncharged polar residues, such as tyrosine, threonine and serine, dominate the antibody-bound complexes [71,72]. Despite the differences in the sequence, comparative analysis of the antibody complexes with the receptor complexes revealed that the interacting segments from the respective dimeric growth factors bear a striking resemblance. The antibodies studied so far seem to span the same expanse on the ligand surface and bring about the neutralizing effect by steric hindrance, not by inducing any conformational change to prevent the receptor from binding to the growth factors.

## Growth factors, signalling and angiogenesis

Angiogenesis, the process of formation of new blood vessels from pre-existing blood vessels, is a vital physiological event in growth and development; it has been implicated in several diseased states and plays a fundamental role in the progression of tumours from their dormant state to their malignant form. Angiogenic molecules, such as those that comprise the VEGF family of cystine-knot growth factors, stimulate ECs to migrate, proliferate and eventually differentiate into new blood vessels through a complex process involving extensive interplay between oncogenes and suppressor genes, stimulatory and inhibitory molecules, proteases and endogenous inhibitors and environmental factors such as the oxygen level (hypoxia) or copper ion. Angiogenesis is such a complex phenomenon that a clear-cut distinction between angiogenic factors as being either inducers or inhibitors would be an oversimplification. Some act as both direct and indirect inducers of the process, while some function contextually, sometimes as inducers and sometimes as inhibitors. It has been experimentally established that the loss of control of termination and stabilization of the blood vessels is caused by the up-regulation of the positive regulators as well as by the exhaustion of the endogenous inhibitors of the process of blood vessel formation.

**Fig. 11:**
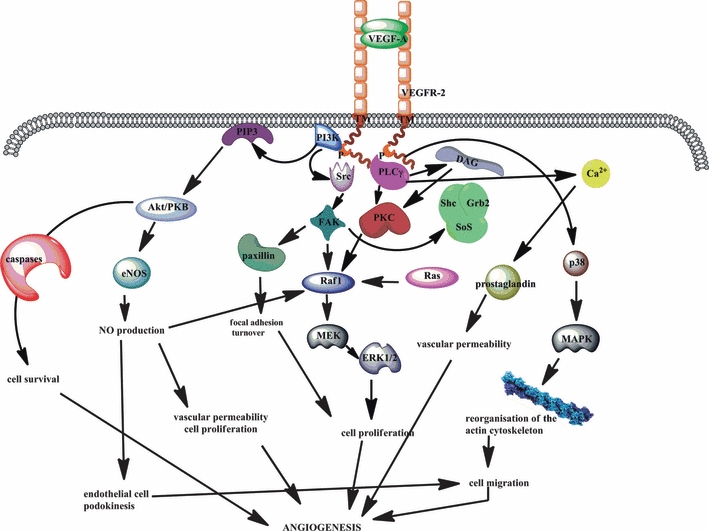
Schematic representation of the molecular players involved in angiogenesis signalling mediated by the interaction of VEGF-A with VEGFR-2 [107]. The text includes all the references that were used to make the figure. DAG, diacyly glycerol; eNOS, endothelial nitric oxide synthase; Grb2, growth factor receptor-bound protein 2; MEK, MAPK/ERK pathway; PKB, protein kinase B; Raf, rapidly accelerated fibrosarcoma; Ras, RAt sarcoma; Sos, son of sevenless.

The ECs are central to the process of blood vessel formation as they integrate a variety of signals arising from growth factors, cell–matrix and cell–cell contacts. It is now an accepted assumption that the critical event in the regulation of the process of angiogenesis is the signal transduction cascade involving members of the VEGF family, especially VEGF-A. Several canonical signalling pathways, such as the MAPK pathway and the phosphatidylinositol 3-kinase (PI3K) pathway are activated by these growth factors as a result of binding to their high-affinity tyrosine kinase receptors and accessory co-receptors, and engagement of molecules such as the integrins and other receptor systems. In contrast to the well-defined role of VEGFR-2 in angiogenic signalling, the function of VEGFR-1 is not as well understood and hence most of the biological responses discussed below pertain to VEGFR-2-mediated signalling (Fig. 11).

A fundamental cellular mechanism by which VEGF maintains blood vessel stability and integrity is by activating anti-apoptotic signalling. VEGF inhibits cellular apoptosis by activating Akt/protein kinase B via a PI3K-dependent pathway [78,79]. VEGF also induces the expression of anti-apoptotic proteins such as Bcl-2 and A1 [80]. These, in turn, inhibit activation of the upstream caspases. Other signalling pathways that promote EC survival include crosstalk between integrins and the VEGF receptors [81], the protein kinase C (PKC) pathway [82] and the association of focal adhesion kinase (FAK) with paxillin. An increase in the degree of phosphorylation of FAK is a point of convergence for diverse EC survival stimuli.

EC proliferation is mainly brought about by the activation of the extracellular signal-regulated kinase (ERK) 1/2 pathway [83,84]. Studies using PKC inhibitors indicate that PKC isoforms (particularly α and ζ) play a crucial role in VEGF mitogenic signalling. PKC-dependent ERK activation is also known to mediate cell proliferation induced by VEGF [85]. VEGF has been shown to induce ras-independent and PKC-mediated induction of the Raf-MAPK/ERK kinase (MEK)-ERK pathway (involving nitric oxide (NO)-mediated Raf-1 activation) to stimulate cell proliferation [86,87].

Cell migration is another key step in the process of angiogenesis. VEGF induces the expression of proteases that promote the degradation of the basement membrane and therefore initiate cell migration. Several experiments point to a critical role for FAK-associated signalling in cell migration [88]. VEGF-induced chemotaxis is also brought about via the p38/MAPK pathway. Activation of the p38 kinase by VEGF leads to reorganization of the actin cytoskeleton, thereby stimulating cell migration [89]. Studies have shown that NO production, stimulated by VEGF, leads to podokinesis of ECs and hence angiogenesis [90,91]. A direct role for NO in VEGF-mediated cell migration came to light when it was shown that Ser1177 of endothelial nitric oxide synthase was phosphorylated via the Akt-dependent pathway [92]. PLC (phospholipase C)-γ activation has also been implicated in mediating cellular responses linked to cell migration stimulated by VEGF [93].

The signalling mechanisms that underlie vascular permeability are not very clearly understood. VEGF induces the formation of fenestrae [94]. These are specialized regions of the plasma membrane that are highly permeable to small solutes. This is a crucial process by which VEGF regulates vascular permeability. VEGFR-2 is the principal signalling pathway for VEGF-mediated increase in vascular permeability during the process of blood vessel formation. Interestingly, unlike the pro-angiogenic VEGFs, the anti-angiogenic VEGFs, such as VEGF_165_b, stimulate vascular permeability by activating VEGFR-1 and not VEGFR-2 [95]. Experiments reveal that VEGF-induced vascular permeability initiates a series of events, including mobilization of intracellular Ca^2+^ [96], Src kinase activation [97] and stimulation of the PI3K [98] and p42/p4MAPK pathways [99]. Ultrastructural studies show that VEGF activation of ECs *in vitro* leads to a very rapid loss of junctional integrity, disassociation of the adherens junctions and actin-dependent contraction of areas of ECs to form transcellular gaps. These changes, which occur as a result of VEGFR signalling, are thought to be regulated by members of the Ras superfamily of small GTPases (Rho/Rac/Cdc42). Rac in particular seems to be linked to the formation of VEGF-induced fenestrae [100].

Stimulation of cellular responses by VEGFR-1 seems to be potentially dependent on the cell type. Only a few functions have been attributed to activation of VEGFR-1, all of which occur in non-ECs, including stimulation of the migration of peripheral blood monocytes by binding to PlGF [101], NO release in trophoblasts [102], up-regulation of matrix metalloproteinases in vascular smooth-muscle cells [103]. Functional expression of VEGFR-1 in adventitial fibroblasts is an important mediator in the pathogenesis of vascular remodelling after arterial injury [104]. It was also shown that VEGFR-1 down-modulates VEGFR-2-mediated EC proliferation via the PI3K-dependent pathways [105]. VEGFR-1-mediated ligand-induced (PlGF-1 or VEGF-A) signal transduction in primary monocytes was also shown to involve two MAPK pathways: p38 and ERK1/2. The activation of these two pathways is strongly dependent on the activation of PI3K [106]. These studies identified the underlying molecular basis for VEGFR-1-mediated signalling in primary monocytes and helped to put VEGFR-1 on the angiogenic signalling map as a functional receptor in its own merit.

## Conclusions and perspectives

The VEGF–VEGFR signalling pathway is considered central to the process of angiogenesis, both in normal growth and development as well as in pathological settings. However, it is clear that the formation of stable and functional blood vessels requires the concerted action of multiple signalling cascades. In this light, new pathways are emerging, both upstream as well as downstream of the VEGF/VEGFR pathway, which are being recognized as essential for normal angiogenesis. Despite the large amount of basic and clinical work that has already been carried out in this area, we are only just beginning to unravel the different functional roles played by the members of VEGF family by virtue of their interactions with the VEGF receptors. There still exist large gaps in our comprehension of the exact mechanism of molecular control of vessel growth and stability by the VEGF/VEGFR system. We need a better understanding of the temporal and spatial orchestration of the different angiogenic signalling pathways to gain a deeper insight into the mechanisms that govern activation of these cystine-knot proteins, specificity of receptor–ligand interactions, co-receptor choice and how these various inputs are integrated at the cellular level to provide the biological response needed. Answers to all these questions will feed towards the design of better drugs/inhibitors targeting VEGFs and the VEGFRs for use in anti-angiogenic therapy. There is still so much to learn about these molecules that angiogenesis research will only intensify further in the years to come.

## Abbreviations

ALS: amyotrophic lateral sclerosis
CCK: cyclic cystine-knot
CNS: central nervous system
CTCK: C-terminal cystine-knot
ECs: endothelial cells
ERK: extracellular signal-regulated kinase
FAK: focal adhesion kinase
GFCK: growth factor cystine-knot
GPH: glycoprotein hormone
HSPG: heparan sulfate proteoglycan
ICK: inhibitor cystine-knot
MAPK: mitogen-activated protein kinase
NGF: nerve growth factor
NO: nitric oxide
NRP: neuropilin
PDGF: platelet-derived growth factor
PI3K: phosphatidylinositol 3-kinase
PKC: protein kinase C
PlGF: placenta growth factor
TGF-β: transforming growth factor-β
VEGF: vascular endothelial growth factor
VEGFR: vascular endothelial growth factor receptor

## References

[1] Murray-Rust J, McDonald NQ, Blundell TL, Hosang M, Oefner C, Winkler F, Bradshaw RA (1993). Topological similarities in TGF-beta 2, PDGF-BB and NGF define a superfamily of polypeptide growth factors. Structure.

[2] Pallaghy PK, Nielsen KJ, Craik DJ, Norton RS (1994). A common structural motif incorporating a cystine knot and a triple-stranded beta-sheet in toxic and inhibitory polypeptides. Protein Sci.

[3] Vitt UA, Hsu SY, Hsueh AJ (2001). Evolution and classification of cystine knot-containing hormones and related extracellular signaling molecules. Mol Endocrinol.

[4] Holbourn KP, Acharya KR, Perbal B (2008). The CCN family of proteins: structure-function relationships. Trends Biochem Sci.

[5] Shibuya M, Yamaguchi S, Yamane A, Ikeda T, Tojo A, Matsushime H, Sato M (1990). Nucleotide sequence and expression of a novel human receptor-type tyrosine kinase gene (flt) closely related to the fms family. Oncogene.

[6] Terman BI, Carrion ME, Kovacs E, Rasmussen BA, Eddy RL, Shows TB (1991). Identification of a new endothelial cell growth factor receptor tyrosine kinase. Oncogene.

[7] Pajusola K, Aprelikova O, Korhonen J, Kaipainen A, Pertovaara L, Alitalo R, Alitalo K (1992). FLT4 receptor tyrosine kinase contains seven immunoglobulin-like loops and is expressed in multiple human tissues and cell lines. Cancer Res.

[8] Neufeld G, Cohen T, Shraga N, Lange T, Kessler O, Herzog Y (2002). The neuropilins: multifunctional semaphorin and VEGF receptors that modulate axon guidance and angiogenesis. Trends Cardiovasc Med.

[9] Stone J, Itin A, Alon T, Pe'er J, Gnessin H, Chan-Ling T, Keshet E (1995). Development of retinal vasculature is mediated by hypoxia-induced vascular endothelial growth factor (VEGF) expression by neuroglia. J Neurosci.

[10] Drake CJ, Little CD (1995). Exogenous vascular endothelial growth factor induces malformed and hyperfused vessels during embryonic neovascularization. Proc Natl Acad Sci USA.

[11] Ferrara N, Chen H, Davis-Smyth T, Gerber HP, Nguyen TN, Peers D, Chisholm V, Hillan KJ, Schwall RH (1998). Vascular endothelial growth factor is essential for corpus luteum angiogenesis. Nat Med.

[12] Dvorak HF, Brown LF, Detmar M, Dvorak AM (1995). Vascular permeability factor/vascular endothelial growth factor, microvascular hyperpermeability, and angiogenesis. Am J Pathol.

[13] Rak J, Filmus J, Finkenzeller G, Grugel S, Marmé D, Kerbel RS (1995). Oncogenes as inducers of tumor angiogenesis. Cancer Metastasis Rev.

[14] Dantz D, Bewersdorf J, Fruehwald-Schultes B, Kern W, Jelkmann W, Born J, Fehm HL, Peters A (2002). Vascular endothelial growth factor: a novel endocrine defensive response to hypoglycemia. J Clin Endocrinol Metab.

[15] Iliopoulos O, Levy AP, Jiang C, Kaelin WGJ, Goldberg MA (1996). Negative regulation of hypoxia-inducible genes by the von Hippel-Lindau protein. Proc Natl Acad Sci USA.

[16] Pal S, Claffey KP, Dvorak HF, Mukhopadhyay D (1997). The von Hippel-Lindau gene product inhibits vascular permeability factor/vascular endothelial growth factor expression in renal cell carcinoma by blocking protein kinase C pathways. J Biol Chem.

[17] Baumgartner I, Isner JM (1998). Stimulation of peripheral angiogenesis by vascular endothelial growth factor (VEGF). VASA.

[18] Backer MV, Hamby CV, Backer JM (2009). Inhibition of vascular endothelial growth factor receptor signaling in angiogenic tumor vasculature. Adv Genet.

[19] Hogan KA, Ambler CA, Chapman DL, Bautch VL (2004). The neural tube patterns vessels developmentally using the VEGF signaling pathway. Development.

[20] Ruhrberg C, Gerhardt H, Golding M, Watson R, Ioannidou S, Fujisawa H, Betsholtz C, Shima DT (2002). Spatially restricted patterning cues provided by heparin-binding VEGF-A control blood vessel branching morphogenesis. Genes Dev.

[21] Schwarz Q, Gu C, Fujisawa H, Sabelko K, Gertsenstein M, Nagy A, Taniguchi M, Kolodkin AL, Ginty DD, Shima DT (2004). Vascular endothelial growth factor controls neuronal migration and cooperates with Sema3A to pattern distinct compartments of the facial nerve. Genes Dev.

[22] Oosthuyse B, Moons L, Storkebaum E, Beck H, Nuyens D, Brusselmans K, Van Dorpe J, Hellings P, Gorselink M, Heymans S (2001). Deletion of the hypoxia-response element in the vascular endothelial growth factor promoter causes motor neuron degeneration. Nat Genet.

[23] Van Den Bosch L, Storkebaum E, Vleminckx V, Moons L, Vanopdenbosch L, Scheveneels W, Carmeliet P, Robberecht W (2004). Effects of vascular endothelial growth factor (VEGF) on motor neuron degeneration. Neurobiol Dis.

[24] Chiappelli M, Borroni B, Archetti S, Calabrese E, Corsi MM, Franceschi M, Padovani A, Licastro F (2006). VEGF gene and phenotype relation with Alzheimer's disease and mild cognitive impairment. Rejuvenation Res.

[25] Wada K, Arai H, Takanashi M, Fukae J, Oizumi H, Yasuda T, Mizuno Y, Mochizuki H (2006). Expression levels of vascular endothelial growth factor and its receptors in Parkinson's disease. Neuroreport.

[26] Olofsson B, Pajusola K, Kaipainen A, von Euler G, Joukov V, Saksela O, Orpana A, Pettersson RF, Alitalo K, Eriksson U (1996). Vascular endothelial growth factor B, a novel growth factor for endothelial cells. Proc Natl Acad Sci USA.

[27] Paavonen K, Horelli-Kuitunen N, Chilov D, Kukk E, Pennanen S, Kallioniemi OP, Pajusola K, Olofsson B, Eriksson U, Joukov V (1996). Novel human vascular endothelial growth factor genes VEGF-B and VEGF-C localize to chromosomes 11q13 and 4q34, respectively. Circulation.

[28] Lagercrantz J, Farnebo F, Larsson C, Tvrdik T, Weber G, Piehl F (1998). A comparative study of the expression patterns for vegf, vegf-b/vrf and vegf-c in the developing and adult mouse. Biochim Biophys Acta.

[29] Bellomo D, Headrick JP, Silins GU, Paterson CA, Thomas PS, Gartside M, Mould A, Cahill MM, Tonks ID, Grimmond SM (2000). Mice lacking the vascular endothelial growth factor-B gene (Vegfb) have smaller hearts, dysfunctional coronary vasculature, and impaired recovery from cardiac ischemia. Circ Res.

[30] Detoraki A, Staiano RI, Granata F, Giannattasio G, Prevete N, de Paulis A, Ribatti D, Genovese A, Triggiani M, Marone G (2009). Vascular endothelial growth factors synthesized by human lung mast cells exert angiogenic effects. J Allergy Clin Immunol.

[31] Li X, Lee C, Tang Z, Zhang F, Arjunan P, Li Y, Hou X, Kumar A, Dong L (2009). VEGF-B: a survival, or an angiogenic factor?. Cell Adh Migr.

[32] Hagberg CE, Falkevall A, Wang X, Larsson E, Huusko J, Nilsson I, van Meeteren LA, Samen E, Lu L, Vanwildemeersch M (2010). Vascular endothelial growth factor B controls endothelial fatty acid uptake. Nature.

[33] Joukov V, Pajusola K, Kaipainen A, Chilov D, Lahtinen I, Kukk E, Saksela O, Kalkkinen N, Alitalo K (1996). A novel vascular endothelial growth factor, VEGF-C, is a ligand for the Flt4 (VEGFR-3) and KDR (VEGFR-2) receptor tyrosine kinases. EMBO J.

[34] Joukov V, Sorsa T, Kumar V, Jeltsch M, Claesson-Welsh L, Cao Y, Saksela O, Kalkkinen N, Alitalo K (1997). Proteolytic processing regulates receptor specificity and activity of VEGF-C. EMBO J.

[35] Stacker SA, Stenvers K, Caesar C, Vitali A, Domagala T, Nice E, Roufail S, Simpson RJ, Moritz R, Karpanen T (1999). Biosynthesis of vascular endothelial growth factor-D involves proteolytic processing which generates non-covalent homodimers. J Biol Chem.

[36] Kaipainen A, Korhonen J, Mustonen T, van Hinsbergh VW, Fang GH, Dumont D, Breitman M, Alitalo K (1995). Expression of the fms-like tyrosine kinase 4 gene becomes restricted to lymphatic endothelium during development. Proc Natl Acad Sci USA.

[37] Oh SJ, Jeltsch MM, Birkenhäger R, McCarthy JE, Weich HA, Christ B, Alitalo K, Wilting J (1997). VEGF and VEGF-C: specific induction of angiogenesis and lymphangiogenesis in the differentiated avian chorioallantoic membrane. Dev Biol.

[38] Yamada Y, Nezu J, Shimane M, Hirata Y (1997). Molecular cloning of a novel vascular endothelial growth factor, VEGF-D. Genomics.

[39] Achen MG, Stacker SA (1998). The vascular endothelial growth factor family; proteins which guide the development of the vasculature. Int J Exp Pathol.

[40] Stacker SA, Caesar C, Baldwin ME, Thornton GE, Williams RA, Prevo R, Jackson DG, Nishikawa S, Kubo H, Achen MG (2001). VEGF-D promotes the metastatic spread of tumor cells via the lymphatics. Nat Med.

[41] Baldwin ME, Halford MM, Roufail S, Williams RA, Hibbs ML, Grail D, Kubo H, Stacker SA, Achen MG (2005). Vascular endothelial growth factor D is dispensable for development of the lymphatic system. Mol Cell Biol.

[42] Haiko P, Makinen T, Keskitalo S, Taipale J, Karkkainen MJ, Baldwin ME, Stacker SA, Achen MG, Alitalo K (2008). Deletion of vascular endothelial growth factor C (VEGF-C) and VEGF-D is not equivalent to VEGF receptor 3 deletion in mouse embryos. Mol Cell Biol.

[43] Rutanen J, Leppänen P, Tuomisto TT, Rissanen TT, Hiltunen MO, Vajanto I, Niemi M, Häkkinen T, Karkola K, Stacker SA (2003). Vascular endothelial growth factor-D expression in human atherosclerotic lesions. Cardiovasc Res.

[44] Jubb AM, Harris AL (2010). Biomarkers to predict the clinical efficacy of bevacizumab in cancer. Lancet Oncol.

[45] Ogawa S, Oku A, Sawano A, Yamaguchi S, Yazaki Y, Shibuya M (1998). A novel type of vascular endothelial growth factor, VEGF-E (NZ-7 VEGF), preferentially utilizes KDR/Flk-1 receptor and carries a potent mitotic activity without heparin-binding domain. J Biol Chem.

[46] Mercer AA, Wise LM, Scagliarini A, McInnes CJ, Büttner M, Rziha HJ, McCaughan CA, Fleming SB, Ueda N, Nettleton PF (2002). Vascular endothelial growth factors encoded by Orf virus show surprising sequence variation but have a conserved, functionally relevant structure. J Gen Virol.

[47] Wise LM, Veikkola T, Mercer AA, Savory LJ, Fleming SB, Caesar C, Vitali A, Makinen T, Alitalo K, Stacker SA (1999). Vascular endothelial growth factor (VEGF)-like protein from orf virus NZ2 binds to VEGFR2 and neuropilin-1. Proc Natl Acad Sci USA.

[48] Wise LM, Ueda N, Dryden NH, Fleming SB, Caesar C, Roufail S, Achen MG, Stacker SA, Mercer AA (2003). Viral vascular endothelial growth factors vary extensively in amino acid sequence, receptor-binding specificities, and the ability to induce vascular permeability yet are uniformly active mitogens. J Biol Chem.

[49] Kiba A, Yabana N, Shibuya M (2003). A set of loop-1 and -3 structures in the novel vascular endothelial growth factor (VEGF) family member, VEGF-ENZ-7, is essential for the activation of VEGFR-2 signaling. J Biol Chem.

[50] Suto K, Yamazaki Y, Morita T, Mizuno H (2005). Crystal structures of novel vascular endothelial growth factors (VEGF) from snake venoms: insight into selective VEGF binding to kinase insert domain-containing receptor but not to fms-like tyrosine kinase-1. J Biol Chem.

[51] Yamazaki Y, Tokunaga Y, Takani K, Morita T (2005). Identification of the heparin-binding region of snake venom vascular endothelial growth factor (VEGF-F) and its blocking of VEGF-A165. Biochemistry.

[52] Maglione D, Guerriero V, Viglietto G, Delli-Bovi P, Persico MG (1991). Isolation of a human placenta cDNA coding for a protein related to the vascular permeability factor. Proc Natl Acad Sci USA.

[53] Ziche M, Maglione D, Ribatti D, Morbidelli L, Lago CT, Battisti M, Paoletti I, Barra A, Tucci M, Parise G (1997). Placenta growth factor-1 is chemotactic, mitogenic, and angiogenic. Lab Invest.

[54] Maglione D, Guerriero V, Viglietto G, Ferraro MG, Aprelikova O, Alitalo K, Del Vecchio S, Lei KJ, Chou JY, Persico MG (1993). Two alternative mRNAs coding for the angiogenic factor, placenta growth factor (PlGF), are transcribed from a single gene of chromosome 14. Oncogene.

[55] Failla CM, Odorisio T, Cianfarani F, Schietroma C, Puddu P, Zambruno G (2000). Placenta growth factor is induced in human keratinocytes during wound healing. J Invest Dermatol.

[56] Hauser S, Weich HA (1993). A heparin-binding form of placenta growth factor (PlGF-2) is expressed in human umbilical vein endothelial cells and in placenta. Growth Factors.

[57] Sawano A, Takahashi T, Yamaguchi S, Aonuma M, Shibuya M (1996). Flt-1 but not KDR/Flk-1 tyrosine kinase is a receptor for placenta growth factor, which is related to vascular endothelial growth factor. Cell Growth Differ.

[58] Park JE, Chen HH, Winer J, Houck KA, Ferrara N (1994). Placenta growth factor. Potentiation of vascular endothelial growth factor bioactivity, in vitro and in vivo, and high affinity binding to Flt-1 but not to Flk-1/KDR. J Biol Chem.

[59] Carmeliet P, Moons L, Luttun A, Vincenti V, Compernolle V, De Mol M, Wu Y, Bono F, Devy L, Beck H (2001). Synergism between vascular endothelial growth factor and placental growth factor contributes to angiogenesis and plasma extravasation in pathological conditions. Nat Med.

[60] Cao Y (2009). Positive and negative modulation of angiogenesis by VEGFR1 ligands. Sci Signal.

[61] Muller YA, Christinger HW, Keyt BA, de Vos AM (1997). The crystal structure of vascular endothelial growth factor (VEGF) refined to 1.93 Å resolution: multiple copy flexibility and receptor binding. Structure.

[62] Iyer S, Leonidas DD, Swaminathan GJ, Maglione D, Battisti M, Tucci M, Persico MG, Acharya KR (2001). The crystal structure of human placenta growth factor-1 (PlGF-1), an angiogenic protein, at 2.0 Å resolution. J Biol Chem.

[63] Iyer S, Scotney PD, Nash AD, Acharya KR (2006). Crystal structure of human vascular endothelial growth factor-B: identification of amino acids important for receptor binding. J Mol Biol.

[64] Leppänen V, Jeltsch M, Anisimov A, Tvorogov D, Aho K, Kalkkinen N, Toivanen P, Ylä-Herttuala S, Ballmer-Hofer K, Alitalo K (2011). Structural determinants of vascular endothelial growth factor-D receptor binding and specificity. Blood.

[65] Pieren M, Prota AE, Ruch C, Kostrewa D, Wagner A, Biedermann K, Winkler FK, Ballmer-Hofer K (2006). Crystal structure of the Orf virus NZ2 variant of vascular endothelial growth factor-E. Implications for receptor specificity. J Biol Chem.

[66] Muller YA, Heiring C, Misselwitz R, Welfe K, Wilfe H (2002). The cystine knot promotes folding and not thermodynamic stability in vascular endothelial growth factor. J Biol Chem.

[67] Wiesmann C, Fuh G, Christinger HW, Eigenbrot C, Wells JA, de Vos AM (1997). Crystal structure at 1.7 Å resolution of VEGF in complex with domain 2 of the Flt-1 receptor. Cell.

[68] Muller YA, Chen Y, Christinger HW, Li B, Cunningham BC, Lowman HB, de Vos AM (1998). VEGF and the Fab fragment of a humanized neutralizing antibody: crystal structure of the complex at 2.4 Å resolution and mutational analysis of the interface. Structure.

[69] Chen Y, Wiesmann C, Fuh G, Li B, Christinger HW, McKay P, de Vos AM, Lowman HB (1999). Selection and analysis of an optimized anti-VEGF antibody: crystal structure of an affinity-matured Fab in complex with antigen. J Mol Biol.

[70] Fuh G, Wu P, Liang W, Ultsch M, Lee CV, Moffat B, Wiesmann C (2006). Structure-function studies of two synthetic anti-vascular endothelial growth factor Fabs and comparison with the Avastin Fab. J Biol Chem.

[71] Leonard P, Scotney PD, Jabeen T, Iyer S, Fabri LJ, Nash AD, Acharya KR (2008). Crystal structure of vascular endothelial growth factor-B in complex with a neutralising antibody Fab fragment. J Mol Biol.

[72] Iyer S, Darley PI, Acharya KR (2010). Structural insights into the binding of vascular endothelial growth factor-B by VEGFR-1(D2): recognition and specificity. J Biol Chem.

[73] Christinger HW, Fuh G, de Vos AM, Wiesmann C (2004). The crystal structure of placental growth factor in complex with domain 2 of vascular endothelial growth factor receptor-1. J Biol Chem.

[74] Leppänen V, Prota AE, Jeltsch M, Anisimov A, Kalkkinen N, Strandin T, Lankinen H, Goldman A, Ballmer-Hofer K, Alitalo K (2010). Structural determinants of growth factor binding and specificity by VEGF receptor 2. Proc Natl Acad Sci USA.

[75] Keyt BA, Nguyen HV, Berleau LT, Duarte CM, Park J, Chen H, Ferrara N (1996). Identification of vascular endothelial growth factor determinants for binding KDR and FLT-1 receptors. Generation of receptor-selective VEGF variants by site-directed mutagenesis. J Biol Chem.

[76] Davis-Smyth T, Presta LG, Ferrara N (1998). Mapping the charged residues in the second immunoglobulin-like domain of the vascular endothelial growth factor/placenta growth factor receptor Flt-1 required for binding and structural stability. J Biol Chem.

[77] Olofsson B, Korpelainen E, Pepper MS, Mandriota SJ, Aase K, Kumar V, Gunji Y, Jeltsch MM, Shibuya M, Alitalo K (1998). Vascular endothelial growth factor B (VEGF-B) binds to VEGF receptor-1 and regulates plasminogen activator activity in endothelial cells. Proc Natl Acad Sci USA.

[78] Gerber HP, McMurtrey A, Kowalski J, Yan M, Keyt BA, Dixit V, Ferrara N (1998). Vascular endothelial growth factor regulates endothelial cell survival through the phosphatidylinositol 3’-kinase/Akt signal transduction pathway. Requirement for Flk-1/KDR activation. J Biol Chem.

[79] Thakker GD, Hajjar DP, Muller WA, Rosengart TK (1999). The role of phosphatidylinositol 3-kinase in vascular endothelial growth factor signaling. J Biol Chem.

[80] Gerber HP, Dixit V, Ferrara N (1998). Vascular endothelial growth factor induces expression of the antiapoptotic proteins Bcl-2 and A1 in vascular endothelial cells. J Biol Chem.

[81] Soldi R, Mitola S, Strasly M, Defilippi P, Tarone G, Bussolino F (1999). Role of alphavbeta3 integrin in the activation of vascular endothelial growth factor receptor-2. EMBO J.

[82] Ilan N, Mahooti S, Madri JA (1998). Distinct signal transduction pathways are utilized during the tube formation and survival phases of in vitro angiogenesis. J Cell Sci.

[83] Wheeler-Jones C, Abu-Ghazaleh R, Cospedal R, Houliston RA, Martin J, Zachary I (1997). Vascular endothelial growth factor stimulates prostacyclin production and activation of cytosolic phospholipase A2 in endothelial cells via p42/p44 mitogen-activated protein kinase. FEBS Lett.

[84] Abedi H, Zachary I (1997). Vascular endothelial growth factor stimulates tyrosine phosphorylation and recruitment to new focal adhesions of focal adhesion kinase and paxillin in endothelial cells. J Biol Chem.

[85] Takahashi T, Ueno H, Shibuya M (1999). VEGF activates protein kinase C-dependent, but Ras-independent Raf-MEK-MAP kinase pathway for DNA synthesis in primary endothelial cells. Oncogene.

[86] Parenti A, Morbidelli L, Cui XL, Douglas JG, Hood JD, Granger HJ, Ledda F, Ziche M (1998). Nitric oxide is an upstream signal of vascular endothelial growth factor-induced extracellular signal-regulated kinase1/2 activation in postcapillary endothelium. J Biol Chem.

[87] Hood JD, Meininger CJ, Ziche M, Granger HJ (1998). VEGF upregulates ecNOS message, protein, and NO production in human endothelial cells. Am J Physiol.

[88] Abedi H, Zachary I (1995). Signalling mechanisms in the regulation of vascular cell migration. Cardiovasc Res.

[89] Rousseau S, Houle F, Landry J, Huot J (1997). p38 MAP kinase activation by vascular endothelial growth factor mediates actin reorganization and cell migration in human endothelial cells. Oncogene.

[90] Noiri E, Hu Y, Bahou WF, Keese CR, Giaever I, Goligorsky MS (1997). Permissive role of nitric oxide in endothelin-induced migration of endothelial cells. J Biol Chem.

[91] Papapetropoulos A, García-Cardeña G, Madri JA, Sessa WC (1997). Nitric oxide production contributes to the angiogenic properties of vascular endothelial growth factor in human endothelial cells. J Clin Invest.

[92] Dimmeler S, Dernbach E, Zeiher AM (2000). Phosphorylation of the endothelial nitric oxide synthase at ser-1177 is required for VEGF-induced endothelial cell migration. FEBS Lett.

[93] Landgren E, Schiller P, Cao Y, Claesson-Welsh L (1998). Placenta growth factor stimulates MAP kinase and mitogenicity but not phospholipase C-gamma and migration of endothelial cells expressing Flt 1. Oncogene.

[94] Roberts WG, Palade GE (1997). Neovasculature induced by vascular endothelial growth factor is fenestrated. Cancer Res.

[95] Glass CA, Harper SJ, Bates DO (2006). The anti-angiogenic VEGF isoform VEGF165b transiently increases hydraulic conductivity, probably through VEGF receptor 1 in vivo. J Physiol (Lond.).

[96] Bates DO, Curry FE (1997). Vascular endothelial growth factor increases microvascular permeability via a Ca(2+)-dependent pathway. Am J Physiol.

[97] Eliceiri BP, Paul R, Schwartzberg PL, Hood JD, Leng J, Cheresh DA (1999). Selective requirement for Src kinases during VEGF-induced angiogenesis and vascular permeability. Mol Cell.

[98] Six I, Kureishi Y, Luo Z, Walsh K (2002). Akt signaling mediates VEGF/VPF vascular permeability in vivo. FEBS Lett.

[99] Wu MH, Yuan SY, Granger HJ (2005). The protein kinase MEK1/2 mediate vascular endothelial growth factor- and histamine-induced hyperpermeability in porcine coronary venules. J Physiol (Lond.).

[100] Eriksson A, Cao R, Roy J, Tritsaris K, Wahlestedt C, Dissing S, Thyberg J, Cao Y (2003). Small GTP-binding protein Rac is an essential mediator of vascular endothelial growth factor-induced endothelial fenestrations and vascular permeability. Circulation.

[101] Clauss M, Weich H, Breir G, Knies U, Rockl W, Wlaternberger J, Risau W (1996). The vascular endothelial growth factor receptor Flt-1 mediates biological activities. Implications for a functional role of placenta growth factor in monocyte activation and chemotaxis. J Biol Chem.

[102] Ahmed A, Dunk C, Kniss D, Wilkes M (1997). Role of VEGF receptor-1 (Flt-1) in mediating calcium-dependent nitric oxide release and limiting DNA synthesis in human trophoblast cells. Lab Invest.

[103] Wang H, Keiser JA (1998). Vascular endothelial growth factor upregulates the expression of matrix metalloproteinases in vascular smooth muscle cells: role of Flt-1. Circ Res.

[104] Jin X, Ge X, Zhu D, Yan C, Chu Y, Chen W, Liu J, Gao P (2007). Expression and function of vascular endothelial growth factor receptors (Flt-1 and Flk-1) in vascular adventitial fibroblasts. J Mol Cell Cardiol.

[105] Zeng H, Sanyal S, Mukhopadhyay D (2001). Tyrosine residues 951 and 1059 of vascular endothelial growth factor receptor-2 (KDR) are essential for vascular permeability factor/vascular endothelial growth factor-induced endothelium migration and proliferation, respectively. J Biol Chem.

[106] Tchaikovski V, Fellbrich G, Waltenberger J (2008). The molecular basis of VEGFR-1 signal transduction pathways in primary human monocytes. Arterioscler Thromb Vasc Biol.

[107] http://www.cambridgesoft.com/software/chembiodraw.

[108] http://www.pymol.org.

[109] Bond CS, Schüttelkopf AW (2009). ALINE: a WYSIWYG protein-sequence alignment editor for publication-quality alignments. Acta Crystallogr.

